# Synergistic Action of Benzyl Isothiocyanate and Sorafenib in a Nanoparticle Delivery System for Enhanced Triple-Negative Breast Cancer Treatment

**DOI:** 10.3390/cancers16091695

**Published:** 2024-04-26

**Authors:** Qi Wang, Nan Cheng, Wei Wang, Yongping Bao

**Affiliations:** Norwich Medical School, University of East Anglia, Norwich NR4 7UQ, UK

**Keywords:** benzyl isothiocyanate, Sorafenib, breast cancer, nanoencapsulation

## Abstract

**Simple Summary:**

In this study, we delve into a challenging aspect of breast cancer known as triple-negative breast cancer (TNBC), which lacks effective treatment options due to its unique characteristics. Our focus is on investigating a novel approach by combining Sorafenib, a drug that targets cancer in several ways, with Benzyl isothiocyanate, a compound with potential cancer-fighting properties. Through our research, we aim to understand how these two treatments work together against TNBC, especially before standard treatment methods are applied. By experimenting with TNBC cell lines and observing the effects of the combined treatment, we seek to uncover a more effective strategy for managing this aggressive form of cancer. Our findings hold the promise of not only enhancing treatment efficacy but also offering new hope for patients battling TNBC, potentially leading to better outcomes and a new direction in the fight against this formidable disease.

**Abstract:**

Triple-negative breast cancer (TNBC) presents a therapeutic challenge due to its complex pathology and limited treatment options. Addressing this challenge, our study focuses on the effectiveness of combination therapy, which has recently become a critical strategy in cancer treatment, improving therapeutic outcomes and combating drug resistance and metastasis. We explored a novel combination therapy employing Benzyl isothiocyanate (BITC) and Sorafenib (SOR) and their nanoformulation, aiming to enhance therapeutic outcomes against TNBC. Through a series of in vitro assays, we assessed the cytotoxic effects of BITC and SOR, both free and encapsulated. The BITC–SOR-loaded nanoparticles (NPs) were synthesized using an amphiphilic copolymer, which demonstrated a uniform spherical morphology and favorable size distribution. The encapsulation efficiencies, as well as the sustained release profiles at varied pH levels, were quantified, revealing distinct kinetics that were well-modeled by the Korsmeyer–Peppas equation. The NP delivery system showed a marked dose-dependent cytotoxicity towards TNBC cells, with an IC50 of 7.8 μM for MDA-MB-231 cells, indicating improved efficacy over free drugs, while exhibiting minimal toxicity toward normal breast cells. Furthermore, the NPs significantly inhibited cell migration and invasion in TNBC models, surpassing the effects of free drugs. These findings underscore the potential of BITC–SOR-NPs as a promising therapeutic approach for TNBC, offering targeted delivery while minimizing systemic toxicity.

## 1. Introduction

Breast cancer remains a leading cause of morbidity and mortality among women worldwide, ranking as the second primary cause of cancer-related deaths following lung cancer [1]. Within the spectrum of breast cancer, triple-negative breast cancer (TNBC) is particularly challenging due to its lack of estrogen and progesterone receptors and HER2 expression, making it unresponsive to most targeted therapies and associated with poorer prognosis and survival rates [2]. In the realm of targeted therapies, Sorafenib (SOR), Nexavar, stands out for its preclinical efficacy against a diverse array of cancer cells, including those from breast [3], melanoma [4], and colorectal origins [5]. Currently, SOR is utilized clinically for treating hepatocellular carcinoma, advanced renal cell carcinoma, and advanced thyroid carcinoma resistant to radioactive iodine, showing promising antitumor activities across various malignancies [6,7,8,9]. Its therapeutic application extends to numerous phase I, II, and III clinical trials, combined with other therapeutics such as with cisplatin plus docetaxel [10,11], paclitaxel [12,13,14], paclitaxel plus carboplatin [15,16] doxorubicin [17], oxaliplatin [18,19], gemcitabine [20,21], capecitabine [8,22], erlotinib [23], vemurafenib [24,25], and Tislelizumab [26].

SOR operates by inhibiting essential intracellular and surface protein kinases such as Vascular Endothelial Growth Factor (VEGFR), Platelet-derived Growth Factor receptor (PDGFR), and Rapidly Accelerated Fibrosarcoma (RAF) kinases, highlighting its broad therapeutic potential [27,28]. Despite its efficacy, studies have raised concerns over SOR’s paradoxical effects in certain contexts, where its antiangiogenic properties might induce hypoxia, thereby enhancing tumor progression and selecting resistant cancer stem cells [29,30]. This highlights the necessity for combinational approaches, incorporating sensitizers to enhance SOR’s therapeutic effect and overcome resistance mechanisms.

Isothiocyanate (ITC), a dietary component found in cruciferous vegetables like broccoli and mustard, emerges as a viable candidate for such combination therapy [31]. Among them, Benzyl ITC (BITC) is one of the best-studied members of the ITC family of compounds. It has attracted a great deal of research interest because of its ability to inhibit chemically induced cancer in animal models [32,33]. BITC inhibits phase I detoxification enzymes but induces phase II enzymes, promotes apoptosis and cell cycle arrest, and inhibits metastasis and angiogenesis [31,34,35,36,37,38,39,40,41]. In addition, BITC was demonstrated to target multiple pathways relevant to cancer cells in combination with other anticancer compounds. Notably, BITC was shown to enhance the efficacy of traditional chemotherapy agents, like cisplatin, in leukemia, lung, oral, and head and neck cancer models [42,43,44,45]. However, the combinatory effects of BITC and SOR on breast cancer cells have yet to be fully elucidated.

The pharmacokinetics of BITC in humans remain underexplored [46]. Known to be metabolized through the mercapturic acid pathway, BITC undergoes conjugation with glutathione (GSH), followed by enzymatic degradation and N-acetylation [47]. Studies in rodents indicate that BITC is rapidly absorbed and excreted [48,49], posing challenges in maintaining therapeutic levels in humans without an effective delivery system. In contrast, the pharmacokinetics of SOR exhibit high variability among individuals [50]. After oral administration, SOR reaches peak plasma concentrations in approximately three hours. It is primarily metabolized in the liver through oxidative processes mediated by CYP3A4, as well as glucuronidation via UGT1A97 [51]. Similar to BITC, SOR is characterized by rapid metabolism leading to a relatively short half-life and variable bioavailability. Additionally, Sorafenib undergoes enterohepatic recycling, further complicating its pharmacokinetic profile. Given these characteristics, there is a critical need for optimized delivery strategies to enhance both BITC and SOR’s efficacy and stability in clinical applications.

Our study aims to explore the potential of BITC to augment the chemotherapeutic efficacy of SOR in breast cancer treatment. By investigating the synergistic effects of BITC and SOR on breast cancer cell lines, we seek to uncover the underlying mechanisms contributing to their enhanced antitumor activity. Furthermore, to optimize the delivery and therapeutic potential of this combination, we developed a nanoparticle (NP)-based co-delivery system, comparing its efficacy with free drugs and their combination in vitro. Our system offers several distinctive advantages over traditional nanoparticle formulations. Firstly, they enhance therapeutic effects by delivering multiple drugs simultaneously, which can produce synergistic outcomes where the combined efficacy surpasses the sum of individual drug effects [33,52]. This multifaceted approach is especially beneficial in managing drug resistance, a common challenge in diseases like cancer where cells may develop resistance to single-drug therapies [52,53]. Additionally, our NPs allow for enhanced drug bioavailability and controlled drug release that can significantly improve treatment effectiveness. These attributes position our nanoparticle delivery system as a promising strategy for the efficient treatment of TNBC.

## 2. Materials and Methods

### 2.1. Materials

Methoxy poly (ethylene glycol)-poly(lactide-co-glycolide) (mPEG-PLGA, monomer feed ratio lactide:glycolide 75:25) was obtained from Jinan Daigang Biotechnology Co. Ltd. (Jinan, China). Sorafenib (catalogue no A3009, CAS# 2884461-73-0, purity 99.89%) was bought from APExBIO (Houston, TX, USA). BITC (catalogue no 89983, CAS# 622-78-6, purity 98.5%), 3-(4,5-Dimethyilthiazol-2-yl)-2,5diphenyltetrayolium bromide (MTT), and dimethyl sulfoxide (DMSO) were obtained from Sigma Aldrich (Dorset, UK). ThinCert™ 24 Well Cell Culture Inserts were bought from Greiner Bio One (Stonehouse, UK). Matrigel was purchased from Corning (Corning Incorporated, New York, NY, USA). Primary antibodies to Cell Division Cycle 2 (Cdc2), p-Cdc2, Checkpoint Kinase 1 (Chk1), cyclinB1, β-actin, fluorescent Alexa 800 goat anti-mice, and Alexa 680 donkey anti-goat Immunoglobulin G (IgG) were all purchased from Santa Cruz Biotechnology (Heidelberg, Germany). Electrophoresis and Western blotting supplies were obtained from Bio-Rad (Hemel Hempstead, UK).

### 2.2. Synthesis and Characterization of BITC–SOR-NPs

BITC and SOR encapsulated NPs (BITC–SOR-NPs) were fabricated using amphiphilic copolymer mPEG-PLGA by an adapted emulsion method as previously described [54]. Briefly, 1 mL Tetrahydrofuran (THF) (with 150 µL ethanol) containing 10 mg of mPEG-PLGA was mixed with 1 mg SOR and/or 1 mg BITC by sonication for 5 min. The solution was then added to 4 mL Poly(vinyl alcohol) (PVA) solution (2%, *w*/*w*) using homogenization and further sonicated for 5 min. The resulting emulsion was then added to 20 mL of PVA solution (0.6%, *w*/*w*) drop-by-drop and further stirred for 30 min. After removing THF through reduced vacuum evaporation, the resulting nanoparticles were obtained by ultra-centrifugation (50,000× *g*, 45 min) and washed with deionized water twice.

The encapsulation efficiencies of SOR and BITC were assessed using high-performance liquid chromatography (HPLC), performed on a Gilson HPLC system (805 MANOMETRIC MODULE). The system configuration included a pump (Gilson 306), mixer (Gilson 811B), loading injector (Model 234 with 20 µL fixed loop), and detector (KNAUER UV detector 2600). Chromatographic analyses were conducted using Clarity 4 software. The separation was achieved on a C18 analytical column (ODS 250 × 4.6 mm internal diameter, 5 µm particle size), maintained at room temperature (25 °C). The mobile phase, composed of water and acetonitrile, flowed at a rate of 1 mL/min, with an injection volume set at 20 µL.

The physical properties of the BITC–SOR-NPs were characterized using two distinct techniques. Nanoparticles were stained with 1% Uranyless (TAAB, London, UK) for enhanced contrast and examined using a Zeiss Gemini 300 SEM (Zeiss, Oberkochen, Germany). Diameter, polydispersity index (PDI), and zeta potential were assessed using dynamic light scattering (DLS) on a Malvern Instruments Ltd. system (Malvern, UK).

### 2.3. In Vitro Drug Release

BITC–SOR-NPs were transferred into a Slide-A-Lyzer MINI Dialysis Device (MWCO 3500 Da, Thermo Fisher Scientific, Altrincham, UK). The dialysis device was then immersed in a Phosphate-buffered saline (PBS) buffer solution (pH 7.4 or pH 5.5) maintained at 37 °C to simulate physiological conditions. At predetermined time intervals, 0.5 mL aliquots were carefully extracted from the surrounding medium for the quantitative analysis of BITC and SOR via HPLC. To maintain sink conditions and consistent volume within the system, each withdrawal was followed by the immediate replacement with an equivalent volume of fresh PBS. The collected release data were analyzed by a non-linear Korsmeyer–Peppas regression model to elucidate the underlying drug release mechanisms from the nanoparticle matrix [55]. Drug transport constants (K) and transport exponents (*n*) of the different liposomal formulations were determined by fitting the in vitro diffusion data to the Korsmeyer–Peppas Equation (1) using the Excel (Microsoft 365) add-in Solver.

(1) $$\frac{Mt}{M\infty }={k\cdot t}^{n}$$

The goodness-of-fit was ascertained by calculating the coefficient of determination (R^2^).

### 2.4. Cell Culture

The human breast epithelial cell line MCF-10A, along with cancer cell lines MCF-7 and MDA-MB-231, were obtained from the American Type Culture Collection (ATCC) (LGC Standards, London, UK). The MCF-7 and MDA-MB-231 were cultured in Dulbecco’s Modified Eagle Medium (DMEM) containing 10% fetal bovine serum (FBS), 2 mM glutamine, penicillin (50 U/mL), and streptomycin (50 mg/mL) (Gibco, Thermo Fisher Scientific, UK) at 37 °C in a humidified atmosphere containing 5% CO_2_. Human normal breast cell line MCF-10A was cultured in DMEM/F12 (Gibco, Thermo Fisher Scientific, UK) supplemented with 5% horse serum (Gibco, Thermo Fisher Scientific, UK), Epidermal growth factor (EGF) (20 ng/mL) (Peprotech, London, UK), Hydrocortisone (0.5 mg/mL) and insulin (10 μg/mL) (Sigma Aldrich, UK) at 37 °C, 5% CO_2_.

### 2.5. In Vitro Cytotoxicity Studies

The cell viability assay was employed to determine the toxicity of SOR, BITC, SOR+BITC and BITC–SOR-NPs towards cultured cells. MCF-7, MDA-MB-231 and MCF-10A cells were seeded into 96-well plates (0.5–1.0 × 10^4^ cells/well). When cells were at 70–80% confluence, different doses of the free drug or NP treatments were added with fresh medium, with DMSO (0.1%) used as control. After 24 or 48 h, the medium was removed, 100 μL (5 mg/mL) MTT solution was added, and the mixture was incubated at 37 °C for 1 h to allow the MTT to be metabolized. The formazan formed was then re-suspended in 100 μL DMSO per well. The final absorbance was recorded using a microplate reader (BMG Labtech Ltd., UK) at a wavelength of 560 nm with a reference wavelength of 650 nm.

### 2.6. Three-Dimensional Spheroid Formation

MDA-MB-231 cells were cultured in Dulbecco’s Modified Eagle Medium (DMEM) containing 10% fetal bovine serum (FBS), 2 mM glutamine, penicillin (50 U/mL), and streptomycin (50 mg/mL) (Gibco, Thermo Fisher Scientific, UK) at 37 °C in a humidified atmosphere containing 5% CO_2_. Three-dimensional (3D) spheroids were obtained using repellent surface multi-well plates. A 25 μL complete medium containing 2 × 10^3^ cells was seeded in each well in an ultra-low attachment 96-well plate. Plates were centrifuged at 290× *g* for 3 min and then incubated at 37 °C. After 24 h, 25 μL of complete medium containing 6 μg/mL collagen I was added to each well, followed by centrifugation of plates at 100× *g* for 3 min. Formed spheroids were then treated with the indicated compounds 48 h later. Treatments were performed by adding 50 μL of fresh complete medium with BITC and/or SOR-free drugs or encapsulated NPs into each well. Images of spheroids were captured using an EVOS M5000 microscope under 4× or 10× magnification and analyzed by ImageJ. The cell viability of MDA-MB-231 spheroids was then measured by resazurin reduction assay. After adding the resazurin solution, the plate was incubated at 37 °C for 6 h and then centrifuged at 290× *g* for 3 min. The fluorescence was read using the microplate reader (BMG Labtech Ltd., Aylesbury, UK) using the following settings: Excitation: 560 nm and Emission: 590 nm.

### 2.7. Colony Formation Assay

MCF-7 and MDA-MB-231 cells were seeded in 6-well plates at 2 × 10^2^ cells/mL and incubated for 24 h. Then, cells were treated with various treatments of SOR, BITC, SOR+BITC, and BITC–SOR-NPs for another 24 h. Cells were maintained for at least 14 days to form colonies. The media was replaced every 3 days. Colonies were then fixed with ice-cold methanol and stained with 0.1% crystal violet for 30 min. Images were analyzed by ImageJ. Results were given as means and standard deviations of three independent experiments with triplicate samples for every treatment condition.

### 2.8. Wound-Healing Assay

MCF-7 and MDA-MB-231 cells were seeded in 12-well plates at 2 × 10^5^ cells/well. After cells reached 100% confluence, scratches were made with a 200 µL pipette tip across the center of the wells without changing the medium. Detached cells were removed by gently washing the well twice with the medium. The wells were then filled with fresh medium containing different treatments. Cells were grown for a further 48 h, while images of the wound area were taken on a Zeiss inverted microscope at 5× magnification. The wound area was quantitatively evaluated using ImageJ. Percentage wound closure was calculated as follows:

(2) $$Wound\;Closure\;\%=\frac{\left(Area\;0-Area\;t\right)}{Area\;0}\times 100\%$$

Area 0 is the wound area from the initial scratch. Area t is the wound area at time t.

### 2.9. Invasion Assay

ThinCert™ Inserts (membrane with 8 µm pore size) were placed in a 24-well cell plate to form an upper chamber and a lower chamber. The upper chamber was then coated with 100 µL Matrigel solution (Matrigel to pre-chilled DMEM 1:10 dilution). MCF-7 or MDA-MB-231 cell suspensions (1 × 10^5^ cells/well) in DMEM medium without FBS were mixed with single or combined BITC and SOR treatments and then added into the upper chambers. In total, 600 µL of 10% FBS-supplemented DMEM medium was placed in the lower chamber. After 24 h 37 °C incubation, non-invading cells were removed by wiping off Matrigel matrix-coating from the upper chamber. The invaded cells on the lower surface of the insert membrane were fixed and stained with 0.1% crystal violet. Images of the invaded cells were taken with an Olympus microscope and further quantified using ImageJ 1.54.

### 2.10. Tube Formation Assay

Human Umbilical Vein Endothelial Cells (HUVECs) (Corning) were used for tube formation, as these tubules were developed from clear elongated cell bodies that connect to form polygon networks. HUVECs were cultured in Endothelial Cell Growth Medium-2 (EGM-2) basal medium (Lonza, Basel, Switzerland) supplemented with EGM-2 supplements (Lonza) and 1% penicillin/streptomycin. Harvested HUVECs were then mixed with conditioned media obtained from MDA-MB-231 after 24 h of exposure to BITC and/or SOR treatments and plated on basement membrane matrix (Corning^®^ Matrigel^®^ Matrix). Next, 2.5 × 10^4^ HUVECs were used for each well and incubated with the conditioned media for 12–16 h at 37 °C. Images of the tube networks were taken by Zeiss microscope and further quantified using ImageJ 1.54 with the Angiogenesis Analyzer plugin [56].

### 2.11. Cell Cycle Analysis

MCF-7 and MDA-MB-231 cells were plated into 12-well plates. After overnight incubation, cells were incubated with SOR, BITC, SOR+BITC, and SOR–BITC-NPs for 24 h. Cells were then harvested and fixed with 80% pre-chilled ethanol at −20 °C overnight. Fixed cells were then stained with BD Pharmingen™ Propidium Iodide/Ribonuclease (PI/RNase) staining buffer and analyzed using a Beckman flow cytometer. For each sample, 10,000 events were collected, and the data were analyzed using CytExpert 1.2 software.

### 2.12. Western Blot Assay

MCF-7 and MDA-MB-231 cells were collected after different treatments, and total cellular protein was extracted. The concentration of protein was measured by a bicinchoninic acid protein assay kit (cat# 23225, Thermo Scientific™). In total, 10–20 μg of proteins were used for Sodium Dodecyl Sulfate Polyacrylamide Gel (SDS-PAGE) electrophoresis and further transferred to 0.22 μm Polyvinylidene Fluoride (PVDF) membranes. After incubating with Anti-Cdc2 p34 antibody (cat# sc-54, Santa Cruz Biotechnology), Anti-p-Cdc2 p34 antibody (cat# sc-136014, Santa Cruz Biotechnology), Anti-Chk1 antibody (cat# sc-8408, Santa Cruz Biotechnology, Santa Cruz, CA, USA), Anti-cyclin B1 antibody (cat# sc-245, Santa Cruz Biotechnology) at 4 °C overnight, these membranes were washed 4 times and incubated with fluorescent secondary antibodies at RT for 1 h. After washing 4 times, the immuno-reactivity was visualized using an LI-COR Odyssey image system. β-actin was chosen as a loading control.

### 2.13. Statistics

All experiments were conducted in triplicate, and results were expressed as the mean ± standard deviation (SD). To determine the half-maximal inhibitory concentration (IC50) values across three cell lines under various treatments, we employed non-linear regression analysis using GraphPad Prism 8 software. Specifically, the “Inhibitor vs. normalized response—Variable slope (four parameters)” model was utilized. This model incorporates the Hill slope, enhancing the precision of dose–response curve modeling and allowing for meticulous adjustments to the curve‘s steepness, thereby providing robust IC50 estimations. The Combination Index (CI) was calculated using CompuSyn 1.0 software, which employs the Chou–Talalay method. In this analysis, a CI value less than 1 indicates synergy between treatments, a CI equal to 1 suggests additive effects, and a CI greater than 1 indicates antagonism.

To assess differences across various treatment groups in our study, we first performed a one-way Analysis of Variance (ANOVA). This initial analysis helped determine if there were statistically significant differences in the dependent variable across the groups. Upon finding significant differences with the one-way ANOVA, we applied Dunnett‘s multiple comparisons test. This post hoc test was specifically used to compare each of the treatment groups against a single control group, thereby identifying which treatments differed significantly from the control. A *p*-value of less than 0.05 was considered statistically significant in all analyses.

## 3. Results

### 3.1. Synthesis of BITC and Sorafenib-Encapsulating Nanoparticles

Over the last two decades, nanoparticle-encapsulated drug delivery systems have demonstrated great advantages over traditional chemotherapy due to their high selectivity toward tumor sites. This delivery strategy is based on the enhanced permeability and retention (EPR) effect, where NPs with a size between 20 and 200 nm can prolong the blood circulation time of the loaded therapeutic agents and preferentially accumulate at tumor sites with reduced nonspecific distribution in healthy tissues. To further enhance the antitumor efficacy and minimize the potential side effects of BITC and SOR combination in clinical usage, we have developed an NP-encapsulated co-delivery system consisting of BITC and SOR for breast cancer treatment.

**Figure 1:**
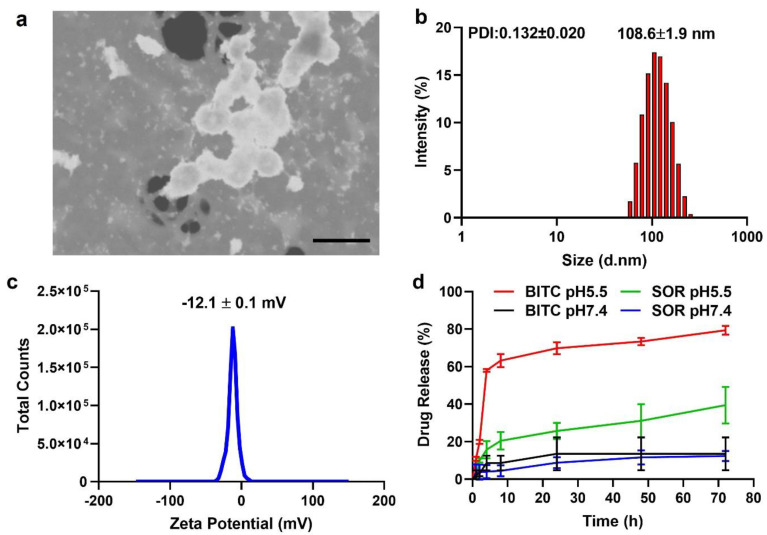
Characterization of BITC−SOR−NPs. (**a**) SEM image of BITC−SOR−NPs. Scale bar, 200 nm. (**b**) Size distribution and Polydispersity index (PDI) of BITC−SOR−NPs measured by dynamic light scattering. (**c**) Zeta potential of BITC−SOR−NPs. (**d**) Release profiles of BITC and SOR at pH 5.5 or 7.4.

Both BITC and SOR in a pre-determined ratio were encapsulated within amphiphilic copolymer methoxy poly(ethylene glycol)-poly(lactide-co-glycolide) (mPEG-PLGA) NPs (termed as BITC–SOR-NPs) according to an adapted emulsion method [54]. The encapsulation efficiencies of BITC and SOR in the BITC–SOR-NPs were 24.0 ± 1.5% and 38.4 ± 6.3%, respectively (Table S1), which gave an approximate molar ratio of 1:1 for BITC/SOR. SEM imaging revealed a uniform spherical morphology of the NPs with a diameter of ~100 nm for the SOR–BITC-NPs (Figure 1a). The DLS results revealed that the hydrodynamic size of BITC–SOR-NPs was ~108 nm with ca. −12.1 mV of the zeta potential (Figure 1b,c), which is an ideal NP size to assist in longer blood circulation and passive tumor targeting. Furthermore, the BITC–SOR-NPs showed good colloidal stability and maintained structural integrity after incubation for 5 days (Figures S5 and S6). Next, the drug release profile of BITC–SOR-NPs was studied at pH 5.5 or 7.4 (Figure 1d). Both BITC and SOR showed sustained release profiles within 48 h and exhibited higher release rates at pH 5.5 than 7.4. To understand the mechanism involved in the release of matrix inside the cell, the release data (pH 5.5) were further analyzed using the Korsmeyer–Peppas model, as described in Equation (1), resulting in good model fits with R^2^ values of 0.99 for BITC and 0.90 for SOR. Calculations from the model fitting revealed that the transport constant (K) for BITC was 8.3, slightly surpassing that for SOR, which was 7.5. Moreover, the release exponent (*n*) was found to be 1.40 for BITIC, indicating a non-Fickian diffusion mechanism possibly dominated by polymer relaxation kinetics, whereas SOR had an *n* value of 0.38, consistent with a Fickian diffusion mechanism [55,57]. These findings delineate a distinct release behavior for each drug, underscoring the impact of combined drug loading on the release dynamics from the nanoparticulate matrix.

### 3.2. Inhibition of TNBC Cell Viability by BITC and SOR

**Figure 2:**
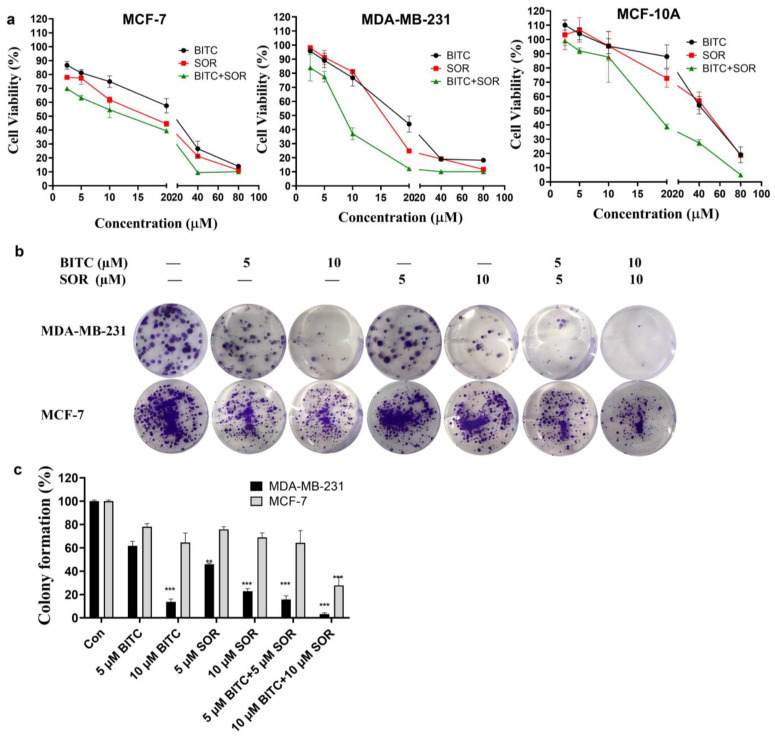
An analysis of the therapeutic efficacy of BITC, SOR, and their combinations. (**a**) Cell viability in MDA-MB-231, MCF-7 and MCF-10A cells was measured using MTT assays 24 h after BITC and SOR treatment. (**b**) A colony formation assay was performed on MDA-MB-231 and MCF-7 cells following treatment with BITC and SOR for 24 h. (**c**) Quantitative analysis of colony formation assay. Data are presented as means ± SD (*n* ≥ 3). Statistical significance is denoted as ** *p* < 0.01, *** *p* < 0.001, compared to the control group, which was treated with 0.1% DMSO.

The effects of BITC and SOR alone and in combination treatment on cell breast cancer cell survival were examined using a pair of well-characterized human breast cancer cell lines (MDA-MB-231 and MCF-7) as models. The MCF-7 cell line, which was isolated from a pleural effusion of stage IV invasive ductal carcinoma, is estrogen receptor (ER)-positive and estrogen-responsive. The MDA-MB-231 cell line is a triple-negative model, which does not express ER, progesterone receptor (PR), and does not have Human Epidermal Growth Factor Receptor 2 (HER-2)/Neu amplification. The MCF-7 cells are aneuploid with high chromosomal instability and partially defective for the G1 and mitotic spindle checkpoint but express normal p53, while MDA-MB-231 cells are partially proficient for all cell cycle checkpoints and express mutant p53 [58]. Selective growth inhibition toward cancer cells is a highly desirable feature of potential cancer preventive and therapeutic agents. In this study, the normal breast cell line MCF-10A was also selected for comparison to determine the antitumor specificity of BITC and SOR on tumor cells. The cell survival of all three cell lines was determined by the MTT cell viability assay, and the results are summarized in Figure 2. Breast cancer MCF-7, MDA-MB-231, and normal breast cells (MCF-10A) were treated with different doses of the drug alone or in combination for 24 h. As shown in Figure 2a, after 24 h treatment, the IC50 of BITC and SOR on MDA-MB 231 cells were 18.65 µM and 15.37 µM, respectively. The IC50 of MCF-7 cells after BITC and SOR treatment were 21.00 µM and 14.25 µM, respectively. These results showed that BITC and SOR alone or in combination inhibited the proliferation of breast cancer cells in a dose-dependent manner. The IC50 of MCF-10A was 43.24 µM and 41.02 µM for BITC and SOR treatment, respectively, after 24 h of incubation, both of which were higher than that of the tumor cell line. MCF-10A was more resistant to growth inhibition by BITC and SOR compared with MDA-MB-231 or MCF-7 cells. Collectively, these results indicated that the human breast cancer cells were significantly more sensitive to growth suppression by BITC and SOR compared with a normal mammary epithelial cell line. Furthermore, we found that the SOR and BITC combination significantly inhibited the viability of both MCF-7 and MDA-MB-231 cells (Figure 2a). BITC largely improved the chemosensitivity of SOR with an IC50 of 8.06 μM for the BITC+SOR group in MDA-MB-231 cells.

To determine whether the combination treatment BITC and SOR had synergistic, additive, or antagonistic effects, Chou–Talalay‘s combination index was used, and CompuSyn was used to calculate the CI. The BITC and SOR combination treated MDA-MB-231 cells showed a synergistic effect with CI values less than 1 at a 1:1 ratio for SOR and BITC at a total concentration of up to 40 µM (Figure S1). In the MCF-7 cell line, the CI values of SOR and BITC at a 1:1 ratio only showed synergistic effects at a total concentration of 5 µM and 80 µM (Figure S2). Also, synergy was observed at a 2:1 ratio of BITC and SOR in MDA-MB-231 cells and a 1:2 ratio of BITC and SOR in MCF-7 cells. As a result of these findings, SOR and BITC can be combined to achieve synergistic effects and inhibit the proliferation of specific types of breast cancer. These results may serve as a basis for the administration of drug combinations for further in vivo study.

In addition, colony formation assays were used to investigate the prolonged inhibitory effect of BITC and SOR combination on breast cancer cells. MDA-MB-231 and MCF-7 cells were treated with different concentrations of BITC and SOR combination to observe the colony formation ability of the cells. As shown in Figure 2b, in the BITC and SOR single-drug treatment groups, the cell colony formation rate of MDA-MB 231 and MCF-7 was decreased with increasing drug concentrations. However, both BITC and SOR inhibited the colony formation of MDA-MB-231 cells better than MCF-7 cells. In addition, when BITC was combined with SOR at low doses of 5 µM and 10 µM, the reduction was even more pronounced than SOR alone (Figure 2c). This indicates that the combination of BITC and SOR can potentially reduce the dose of SOR used, alleviate side effects in patients, or kill more breast tumor cells with the same dose of SOR.

### 3.3. Inhibition of TNBC Cell Migration and Invasion by BITC and SOR

**Figure 3:**
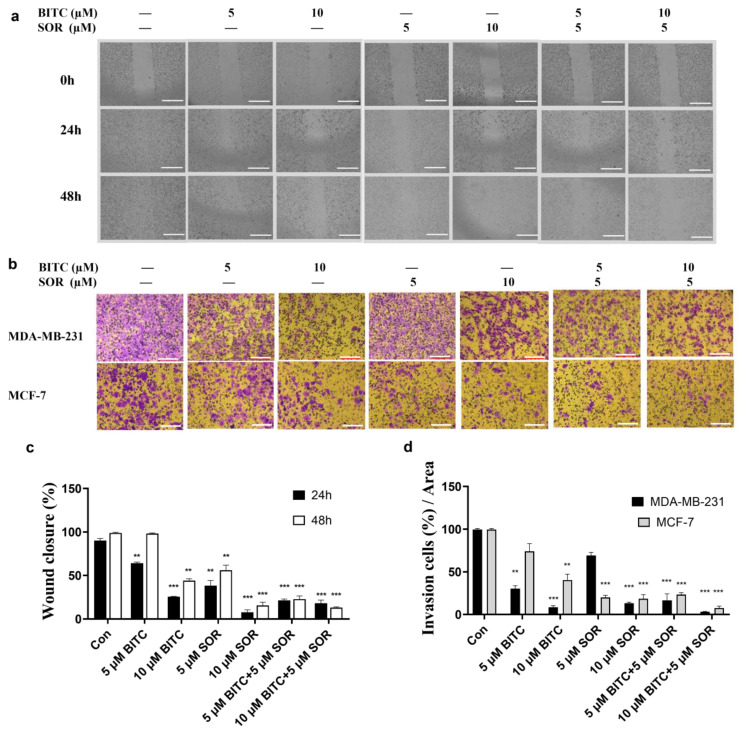
BITC and SOR combinational treatment effects on cell invasion and migration in breast cancer cells MDA-MB-231 and MCF-7: (**a**) Migration was measured by wound-healing assay for MDA-MB-231 and MCF-7 cells after different treatments for up to 48 h, scale bar 1000 µm; (**b**) Invasion assay for MDA-MB-231 cells after BITC and/or SOR treatments for 24 h, scale bar 100 µm; (**c**) Quantitative analysis of wound closure; (**d**) Quantitative analysis of cell invasion. Data are presented as means ± SD (*n* = 3). Statistical significance is denoted as ** *p* < 0.01, *** *p* < 0.001, compared to the control group.

The combination of BITC and SOR significantly decreased the migration and invasion of breast cancer cells. The invasion and migration ability of tumor cells plays an important role in the migration of tumor cells to peripheral tissues. To analyze the effect of BITC and SOR on breast cancer cell migration, a wound-healing assay was performed on the confluent monolayers of MDA-MB-231 cells. BITC and SOR alone and in combination were tested up to 10 µM. As shown in Figure 3a,c, BITC and SOR treatment inhibited MDA-MB-231 migration into the wound in a time- and dose-dependent manner, shown as the increased wound area compared to the control. Furthermore, compared to each drug alone, exposure of MDA-MB-231 cells to BITC and SOR combination for 24 h resulted in statistically significant inhibition of wound closure (*** < 0.001), which indicates the migration inhibition by the combination treatment is more effective than BITC and SOR alone.

A Matrigel-coated Transwell assay was used to investigate the ability of BITC and SOR to inhibit cancer cell invasion. Both MDA-MB-231 and MCF-7 cells were treated with different concentrations of BITC and SOR. Results showed that cell invasion was decreased in a dose-dependent manner (Figure 3b,d). The 10 µM BITC or SOR alone significantly reduced cancer cell invasion vs. the control group in both cell lines. Among all treatment groups, the 5 µM SOR and 10 µM BITC combination was the most effective group, which agreed with the migration assay. The result showed that the combination of low concentration of BITC and SOR can achieve the same inhibition efficacy as high concentration of SOR treatment, which indicates that the combination of BITC and SOR can reduce the dose of SOR administration. It is also interesting to see that cell invasion was reduced more in MDA-MB-231 by BITC treatment, while SOR inhibited more of the MCF-7 cell invasion. These results indicate that different subtypes of breast cancer might respond differently to BITC and SOR.

### 3.4. Inhibition of TNBC Tube Formation by BITC and SOR

**Figure 4:**
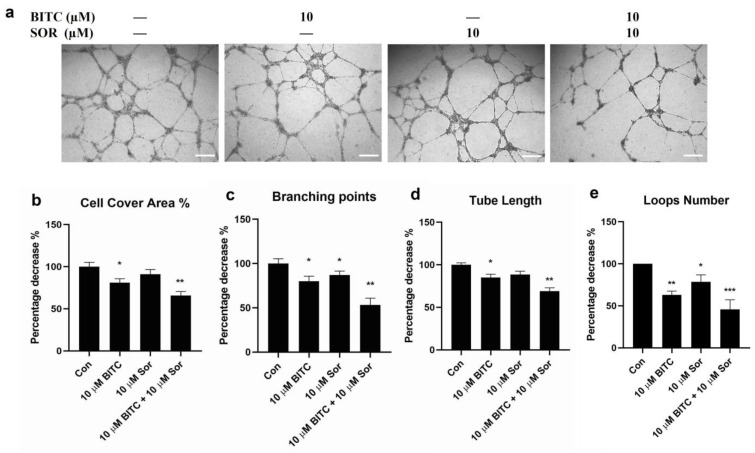
Effects of BITC and SOR combinational treatment on inhibition of angiogenesis in breast cancer cells MDA-MB-231: (**a**) Angiogenesis was measured by tube formation assay for MDA-MB-231 cells after different treatments up to 24 h (scale bar 200 µm); (**b**–**e**) Quantitative analysis of tube formation characteristics. Data are presented as means ± SD (*n* = 3). Statistical significance is denoted as * *p* < 0.05, ** *p* < 0.01, *** *p* < 0.001, compared to the control group.

In vitro endothelial tube formation assays provide a model for studying the differentiation and modulation of endothelial tube formation by potential anticancer agents. To investigate the pro-angiogenic effects of BITC and/or SOR, a tube formation assay was performed using HUVECs to observe vascular network formation. The results showed that at 16 hours, all four network characters, including cell cover area, total branching point, total tube length, and loop number (Figure 4) by conditioned media obtained from 10 µM BITC treatment, were significantly longer than those in the control group. Although the cell cover area and total tube length in the 10 µM SOR conditioned media group were lower than that in the control group, there was no statistical difference between them. The combined treatment of BITC and SOR is most effective in reducing the number of branching points and loops by 50% compared to the control.

### 3.5. Induction of Cell Cycle Arrest by BITC and SOR in TNBC Cells

**Figure 5:**
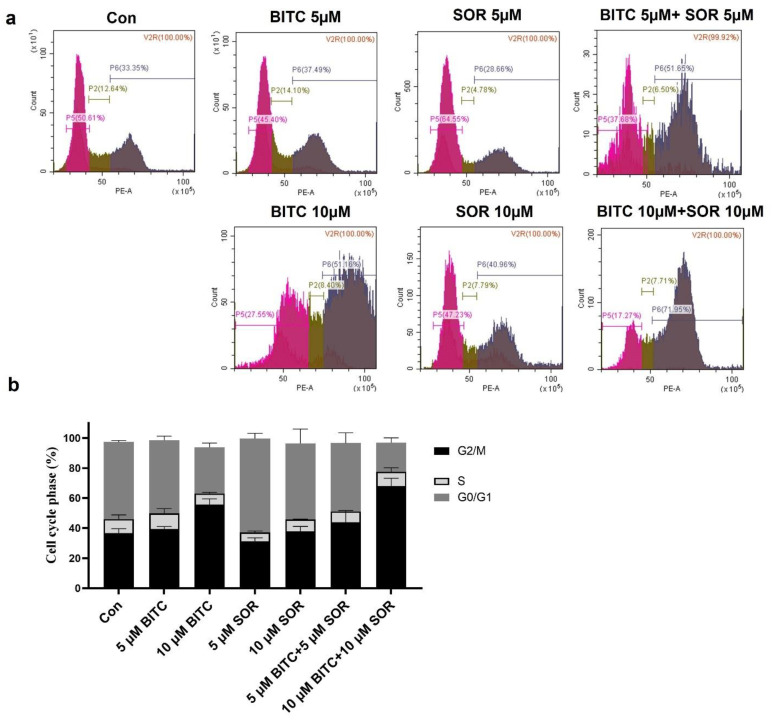
Effects of BITC and SOR combined treatment on MDA-MB-231 cell cycle: (**a**) Flow cytometry analysis of the cell cycle distribution after 24 h of treatment with BITC and/or SOR; (**b**) Quantitative evaluation of cells arrested at G2. Data are presented as means ± SD (*n* = 3).

To study the combinational effect of BITC and SOR on cancer cell growth inhibition, we examined their effects on cell cycle distribution by flow cytometry following staining with PI (Figure 5a). Representative histograms for cell cycle distribution in MDA-MB-231 cells following a 24 h exposure to BITC and/or SOR are shown in Figure 5b. Exposure of MDA-MB-231 cells with up to 10 µM BITC for 24 h resulted in significant enrichment of the G2-M fraction, which was accompanied by a decrease in mainly G0-G1 and S phase cells. In addition, the BITC and SOR combination caused a ∼2-fold increase in the G2-M fraction compared with the DMSO-treated control. Like MDA-MB-231, BITC and SOR treatment caused a statistically significant increase in the G2-M fraction of MCF-7 cells (Figure S3). However, the combination treatment mediated G2-M phase cell cycle arrest was relatively more pronounced in MCF-7 cells than in MDA-MB-231 cells, as the treatment caused a ∼3-fold increase in the percentage of G2-M fraction over control in MCF-7 cells (Figure S3). BITC (5 μM) or SOR (5 μM) alone did not produce a significant effect on G2-M phase arrest, while BITC (5 μM) + SOR (5 μM) could increase G2-M phase arrest to ~60%. This confirms the synergistic effect of BITC and SOR in cell cycle arrest. These results indicated that the BITC and SOR combination-mediated inhibition of MDA-MB-231 and MCF-7 cell survival was probably associated with a sustained G2-M phase cell cycle arrest.

### 3.6. Inhibition of TNBC 3D Spheroids Growth by BITC and SOR

**Figure 6:**
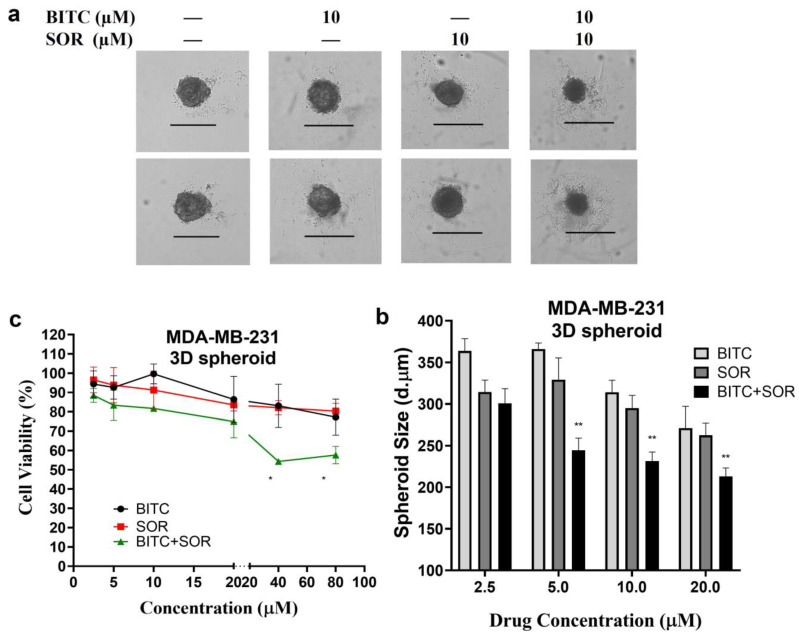
Effects of BITC and SOR combined treatment on MDA-MB-231 spheroids: (**a**) MDA-MB-231 cells were seeded for spheroid generation, and brightfield images of spheroids after 48 h BITC and/or SOR treatments were captured using an EVOS M5000 microscope under 4× magnification (Scale bar denotes 500 μm); (**b**) Size comparison of MDA-MB-231 spheroids measured by ImageJ and expressed as the diameter of the Spheroid; (**c**) The cell viability of MDA-MB-231 spheroids was measured by resazurin reduction assay. Data are presented as means ± SD (*n* = 3). Statistical significance is denoted as * *p* < 0.05, ** *p* < 0.01, compared to the control group, which was treated with 0.1% DMSO.

The tumor 3D spheroid model in vitro is effective at detecting malignant cells and tumorigenesis, as well as assessing drug resistance. Spheroids provide a more accurate representation of the complex cellular environment in vivo compared to monolayer culture, which has been extensively studied in cancer research. In fact, 3D culture systems provide unique opportunities to culture cancer cells alone or in combination with other cell types in a spatially appropriate manner, and they encourage cell–cell and cell–matrix interactions that mirror the cancer environment [59]. This interaction leads to the 3D-cultured cells acquiring morphological and cellular properties similar to those of tumors in vivo. Among various 3D culture systems, spheroids are the most extensively characterized and widely used models [60]. Research has shown that spheroids of breast cancer, such as MCF-7 spheroids [61], reveal the role played by the microenvironment in tumor progression and are more resistant to drug treatments than those cultured as a 2D monolayer. In the current study, the MDA-MB-231 cells were assembled into spheroids in low attachment microwell plates. According to brightfield images of MDA-MB-231 spheroids, a round and compact structure with a well-defined outer perimeter was observed. As shown in Figure 6, the MDA-MB-231 cells in spheroids grew more slowly following treatment with BITC and/or SOR. In addition, combining both BITC and SOR treatments significantly reduced the size of the spheroids. Resazurin reduction assays were performed instead of MTT analysis in order to evaluate the viability of cells derived from MDA-MB-231 spheroids. Viable cells with active metabolism can reduce resazurin into the water-soluble resorufin product, which is pink and fluorescent. It minimizes the disturbance of spheroids during the assay. It was demonstrated in this study that exposure to either SOR or BITC reduced the viability of MDA-MB-231 spheroids in a concentration-dependent manner. Moreover, spheroids are more resistant to all treatments than monolayers of cells.

### 3.7. Induction of Cell Cycle Arrest via Inhibition of Cyclin B1, Chk1, and Cdc2 in TNBC Cells

**Figure 7:**
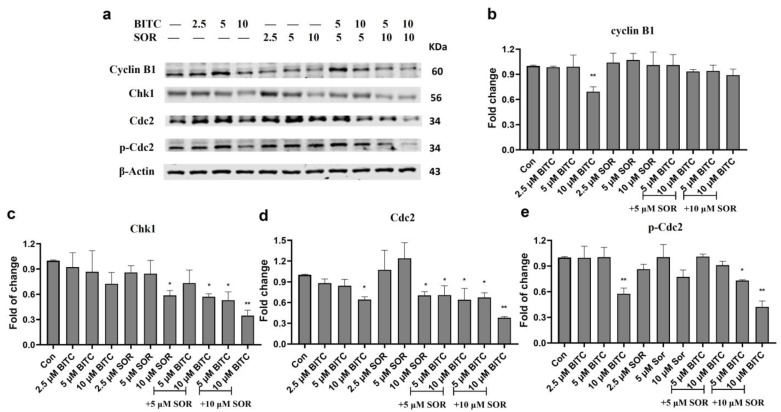
Western Blot analysis of the effects of BITC and SOR treatment on G2 phase protein expression in MDA-MD-231 cells. (**a**) Expression of cyclin B1, Chk1, Cdc2, and p-Cdc2 proteins after BITC and SOR treatment for 24 h. Band densitometric analysis of cyclin B1 (**b**), Chk1 (**c**), Cdc2 (**d**), and p-Cdc2 (**e**) protein expression. β-actin served as a loading control. Band densities were normalized against β-actin, and results were expressed as fold changes relative to controls. Data are presented as means ± SD (*n* = 3). Statistical significance is denoted as * *p* < 0.05, ** *p* < 0.01, compared to the control group. Original western blots are presented in File S1.

The combination of BITC and Sorafenib induced cell cycle arrest by inhibiting cyclin B1, Chk1, and Cdc2. To investigate the mechanism of BITC and SOR-mediated cell cycle arrest, we examined their effects on levels of protein expression associated with G2 phase regulation, including cyclin B1, Cdc2, which is also known as Cyclin Dependent Kinase 1 (CDK1), and Chk1, using Western blotting, as shown in Figure 7 (MDA-MB-231) and Figure S4 (MCF-7). As shown in Figure 7, cyclin B1 expression was only significantly down-regulated in MDA-MB-231 by 10 µM BITC treatment. However, in MCF-7 cells, cyclin B1 expression was significantly down-regulated by both SOR treatment alone and BITC+SOR combination treatment. In MDA-MB-231 cells, both Cdc2 and Chk1 expression was significantly down-regulated by the combination treatment. BITC (5 μM) or SOR (5 μM) alone did not produce a significant effect on the expression of Cdc2, but BITC (5 μM) + SOR (5 μM) could suppress Cdc2 expression to 30%. A similar trend was seen in the combination treatment inhibition of Chk1 expression in both MDA-MB-231 and MCF-7 cells. Phosphorylated Cdc2 was significantly down-regulated by 10 μM BITC alone or BITC and SOR combinations in MDA-MB-231 cells, while MCF-7 seems more sensitive towards BITC and SOR treatment as BITC (5 μM) + SOR (5 μM) could suppress the level of phosphorylated Cdc2 to 30%.

### 3.8. Enhanced Anticancer Efficacy of BITC–SOR-NPs in TNBC Cells and 3D Spheroids

**Figure 8:**
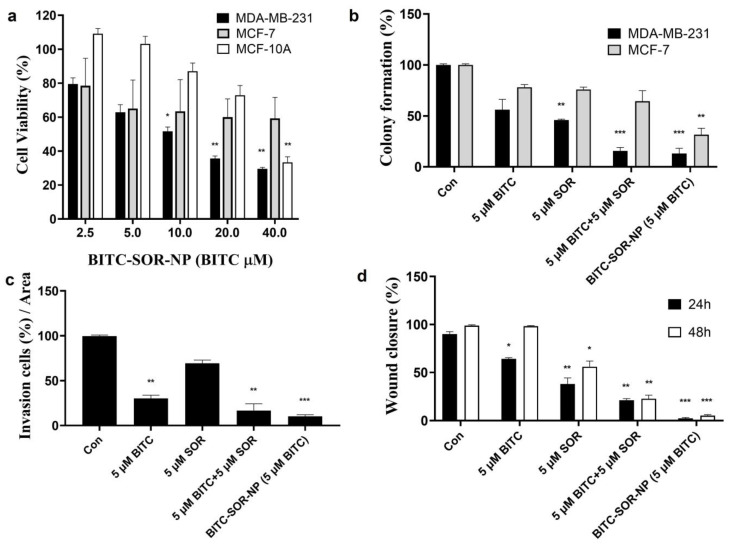
Effects of BITC–SOR-NPs treatment on breast cancer cell lines: (**a**) Cytotoxicity of MDA-MB-231, MCF-7, and MCF-10A cells after different treatments for 24 h; (**b**) Colony formation of MDA-MB-231, MCF-7, and MCF-10A cells after different treatments for 24 h; (**c**) The quantitative results of invasion assay for MDA-MB-231 cells after different treatments for 24 h; (**d**) Migration study of MDA-MB-231 cells after different treatments for 24 h (quantitative results from wound-healing assay). Data are presented as means ± SD (*n* = 3). Statistical significance is denoted as * *p* < 0.05, ** *p* < 0.01, *** *p* < 0.001, compared to the control group.

The therapeutic efficacy of BITC–SOR-NPs was investigated and compared with free drugs in vitro. MDA-MB-231, MCF-7 and MCF-10A cells were seeded into 96-well plates. Twenty-four hours later, the cells were incubated with BITC–SOR-NPs at different concentrations for another 24 h. The cell viability was analyzed using an MTT assay. As shown in Figure 8a, BITC–SOR-NPs exhibited clear dose-dependent toxicity towards MDA-MB-231 cells. Encouragingly, in MDA-MB-231 cells, BITC–SOR-NPs achieved the most significant cytotoxicity with an IC50 as low as 7.8 μM, which is attributed to its enhanced cellular uptake compared with the free drug. There was no significant cytotoxicity from the BITC–SOR-NPs in the normal breast cell line MCF-10A (up to 40 μM). The results suggested in comparison with MCF-7 and MCF-10A cells, BITC–SOR-NPs significantly inhibit the growth of triple-negative breast cancer MDA-MB-231 cells even at low doses (10 μM), and such selectivity was enhanced by nano-encapsulation. These findings were further confirmed by the colony formation assay, where BITC–SOR-NPs showed stronger inhibition of colony formation in MDA-MB-231 cells than in MCF-7 cells (Figure 8b). BITC–SOR-NPs were also evaluated for their effects on breast cancer cell migration and invasion quantitatively using wound closure assays and Transwell invasion assays (Figure 8c,d). The results demonstrated that a combination of BITC and SOR free drugs reduced cell migration and invasion by 20%. In contrast, BITC–SOR-NPs showed a maximal inhibition of cell migration and invasion of over 90%. The BITC–SOR-NPs achieved a greater inhibition of cell migration and invasion than free BITC and SOR, probably due to the more efficient uptake of the NPs into the cell. The effects of BITC–SOR-NPs on MDA-MB-231 spheroids were also examined, and the results are shown in Figure S7. Unlike the free drug combination, BITC–SOR-NPs significantly inhibit cell viability of spheroid cells at a much lower concentration of 10 μM.

## 4. Discussion

The therapeutic landscape of breast cancer, particularly TNBC, presents significant challenges due to its heterogeneity and lack of targeted therapies. This study explored the combined effects of BITC and SOR on the viability, invasion, migration, and cell cycle arrest of breast cancer cell lines MDA-MB-231 and MCF-7, contributing to the identification of potential synergistic approaches for cancer therapy.

Our findings demonstrated a significant inhibitory effect of BITC and SOR, both individually and in combination, on the proliferation of breast cancer cells. This is in line with previous research that identified BITC‘s potential to induce apoptosis and inhibit cell growth across various cancer types through mechanisms such as oxidative stress induction and the modulation of apoptosis-related proteins [44,62,63]. Similarly, Sorafenib‘s effectiveness in targeting multiple kinases involved in tumor cell proliferation and angiogenesis is well-documented, with its role in inhibiting the RAF/MEK/ERK pathway contributing to its therapeutic efficacy in cancers such as renal cell carcinoma and hepatocellular carcinoma [64,65,66].

Interestingly, our study found that the combination of BITC and SOR at specific doses significantly reduced the viability of breast cancer cells more effectively than either agent alone. This suggests a synergistic interaction, potentially offering a strategic advantage by lowering the effective dose required for each drug, thereby reducing associated toxicities. The use of combination therapies in cancer treatment is supported by their ability to target multiple pathways simultaneously, a strategy that is particularly relevant for complex diseases like cancer, where redundancy and compensatory mechanisms can quickly lead to drug resistance [67]. The observed synergistic growth inhibition effect of BITC and SOR at a 1:1 molar ratio towards MDA-MB-231 cells highlights the strategic benefit of combining these compounds. This synergy likely arises from the concurrent targeting of multiple pathways involved in cell proliferation, apoptosis, and metastasis, thereby thwarting the cancer cell’s ability to adapt and develop resistance. The enhanced sensitivity of MDA-MB-231 cells compared to the normal breast cell line MCF-10A underscores the therapeutic window offered by this combination, allowing for effective cancer targeting with minimal impact on healthy tissues.

Moreover, our investigation into the mechanism of action revealed that the combination treatment notably induced G2/M phase arrest, an effect that could be attributed to the disruption of the cell cycle, which is a critical control mechanism for cellular proliferation and a common target in cancer therapy [68]. It is well known that cell cycle progression involves sequential activation of Cyclin-dependent kinases whose activation is dependent on their association with regulatory cyclins [69]. A complex formed by the association of Cdc2 with cyclin B1 plays a major role in the regulation of G2-M transition. Our results have demonstrated that the BITC-mediated inhibition of G2-M progression in MDA-MB-231 cells is associated with a decrease in the protein levels of Cdc2, cyclin B1, and Chk1, while SOR alone does not significantly affect those protein levels. However, BITC and SOR, in combination, synergistically inhibit cell cycle progression from S to G2 phase through down-modulation of Cdc2, cyclin B1, and Chk1. This result is in agreement with previous studies showing that dietary ITCs combined with other anticancer agents synergistically inhibit cell cycle progression from S to G2 phase [70,71]. It is reasonable to postulate that the BITC and SOR combination-mediated cell cycle arrest in MDA-MB-231 cells is likely due to the inhibition of complex formation between Cdc2 and cyclin B1.

Nanoparticle encapsulation offers a multifaceted advantage in drug delivery, primarily through improved solubility, stability, and bioavailability of the encapsulated agents. Furthermore, NPs can be engineered to exhibit passive targeting capabilities by exploiting the enhanced permeability and retention (EPR) effect seen in tumor vasculature, thereby increasing drug accumulation at the tumor site while minimizing systemic exposure and associated toxicities [33,72]. In the context of BITC and SOR, encapsulation within nanoparticles could potentially address any solubility issues, ensuring a more controlled and sustained release of the drugs, thus maintaining therapeutic concentrations within the tumor microenvironment for an extended period.

The destruction of breast cancer cells by our NP system could involve both enhanced drug delivery mechanics and potentiated pharmacological actions. The nanoparticles are engineered to optimize the cellular uptake of BITC and SOR, facilitating more direct and efficient drug delivery into the cancer cells. Once inside, the controlled release properties of the NPs ensure sustained drug availability, maintaining therapeutic concentrations that continuously engage the targeted pathways over extended periods. This sustained interaction significantly enhances the drugs’ abilities to induce apoptosis and disrupt cell signaling pathways critical for cancer cell survival and proliferation. For instance, the NP system enhances the delivery of BITC into the cells, where it effectively modulates key apoptosis-regulating proteins and stress response pathways, while SOR‘s ability to inhibit kinase pathways is potentiated by its increased intracellular concentration. This leads to a more pronounced and sustained induction of cell cycle arrest and apoptosis than either drug could achieve alone.

The enhanced treatment efficiency observed in 3D cell model analysis with NP encapsulation of BITC and SOR over free drug administration is particularly noteworthy. Three-dimensional cell culture models more accurately replicate the complex structure, function, and microenvironment of tumors in vivo, offering a more predictive platform for evaluating the efficacy of nanoparticle-delivered therapies [73]. NPs can penetrate the extracellular matrix and reach cancer cells more effectively in 3D models, providing a uniform distribution of therapeutic agents throughout the tumor spheroids. This improved penetration and distribution can lead to enhanced anticancer effects, as demonstrated by the superior performance of NP-encapsulated BITC and SOR in our study.

## 5. Conclusions

In conclusion, our investigation into the combinatory effects of BITC and SOR on human breast cancer cell viability has revealed significant findings. Notably, a 1:1 molar ratio of BITC and SOR presents a synergistic inhibition of growth in MDA-MB-231 cells, whereas a 1:2 ratio is optimal for MCF-7 cells. This combination treatment exhibits preferential toxicity toward cancerous cells over normal breast cells, a distinction that is notably amplified when these compounds are encapsulated within nanoparticles, particularly evident in 3D spheroid models. The observed synergistic inhibition is mechanistically linked to the arrest of the cell cycle in the G2-M phase, mediated by the down-regulation of cyclin B1 and Cdc2. Collectively, these findings underscore the potential of the BITC and SOR combination as a foundation for crafting innovative, highly effective therapies for managing triple-negative breast cancer. The use of nanoparticle (NP) encapsulation to co-deliver BITC and SOR not only enhances the drug combination sensitivity but also significantly boosts therapeutic efficacy, marking a promising advance in cancer treatment strategies.

## Data Availability

Data will be made available on request.

## Supplementary Materials

The following supporting information can be downloaded at: https://www.mdpi.com/article/10.3390/cancers16091695/s1, Figure S1: Chou-Talalay’s Combinational Index (CI) was calculated using cell viability assay value in MDA-MB-231 cells treated with BITC and SOR combinations. (a) Combination Index Plot of BITC and SOR combination treatment. (Total Dose: dose of BITC plus dose of SOR; Fa: average effect values; CI value: combinational index value. CI values for synergism is 0–1, and for antagonism is 1–∞) (b) Cell viability of MDA-MB-231 cells was measured using MTT assays 24 h after BITC and SOR treatment; Figure S2: Chou-Talalay’s Combinational Index (CI) was calculated using cell viability assay value in MCF-7 cells treated with BITC and SOR combinations. (a) Combination Index Plot of BITC and SOR combination treatment. (Total Dose: dose of BITC plus dose of SOR; Fa: average effect values; CI value: combinational index value. CI values for synergism is 0-1, and for antagonism is 1–∞) (b) Cell viability of MCF-7 cells was measured using MTT assays 24 h after BITC and SOR treatment; Figure S3: Effects of BITC and SOR combined treatment on MCF-7 cell cycle. (a) Flow cytometry analysis of the cell cycle distribution after 24 h of treatment with BITC and/or SOR. (b) Quantitative evaluation of cells arrested at G2. Data are presented as means ± SD (*n* = 3). * *p* < 0.05, ** *p* < 0.01, *** *p* < 0.001. Figure S4: Effects of BITC and SOR treatment on G2 phase protein expression in MCF-7 cells. 24 h after treatment, the expression of cyclin B1, Chk1, Cdc2, and p-Cdc2 proteins was analyzed by Western blot. Expression of β-Actin served as a loading control. Band densities were normalized against β-actin, and results were expressed as fold changes relative to controls. Data are presented as means ± SD (*n* = 3). * *p* < 0.05, ** *p* < 0.01. Figure S5: BITC and SOR encapsulated nanoparticle size stability in various solutions. (a) dH2O, (b) PBS, (c) DMEM+10%FBS. The size of nanoparticles was measured by dynamic light scattering; Figure S6: BITC and SOR encapsulated nanoparticle Polydispersity index (PDI) stability in various solutions. (a) dH2O, (b) PBS, (c) DMEM+10%FBS. The PDI of nanoparticles was measured by dynamic light scattering; Figure S7: Effects of BITC-SOR-NPs treatment on 3D spheroid of MDA-MB-231 spheroids. (a) Cytotoxicity of MDA-MB-231 spheroids after BITC and/or SOR free drugs or encapsulated NP treatments for 4 days. Representative images of MDA-MB-231 spheroids were captured using EVOS M5000 microscope under 10× magnification brightfield image, nuclear (blue Hoechst dye staining) and dead (red EthD-1 dye staining) cell population. (b) Fluorescence intensity of EthD-1 dye staining on MDA-MB-231 spheroids after BITC and/or SOR treatments. (c) and (d) Cell viability study of MDA-MB-231 spheroids after 48 h treatments of BITC-SOR-NPs and free drug combination. Data are presented as means ± SD (*n* = 3). * *p* < 0.05; Table S1: Table of PLGA BITC/Sorafenib NP characteristics. File S1: Original western blots.
