# Supplementary material for: Synergistic Action of Benzyl Isothiocyanate and Sorafenib in a Nanoparticle Delivery System for Enhanced Triple-Negative Breast Cancer Treatment
Source: Cancers (Basel). 2024 Apr 26;16(9):1695. doi: 10.3390/cancers16091695 (PMC11083210; doi:10.3390/cancers16091695)

MDA-MB-231

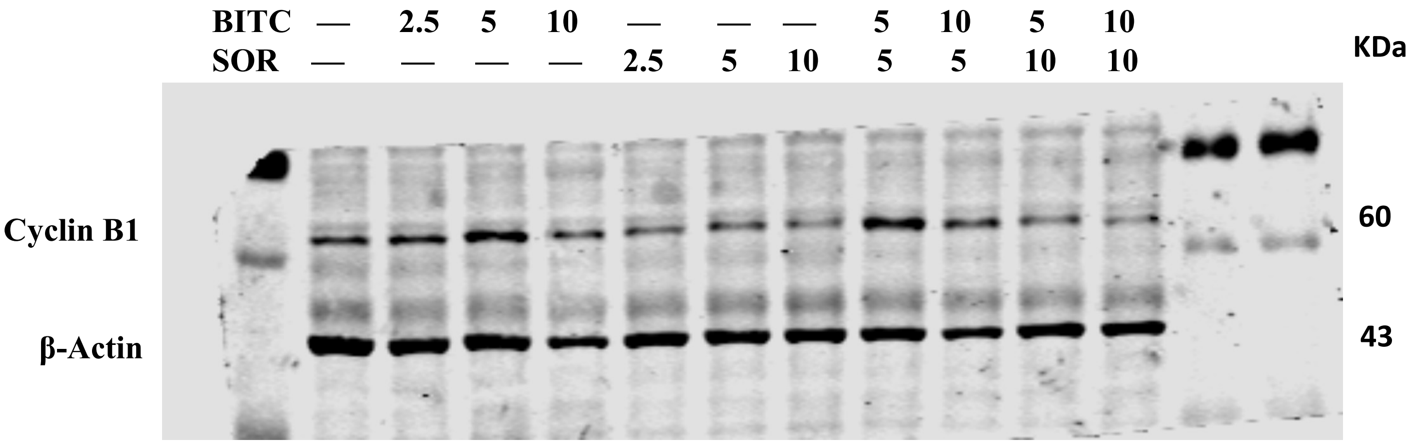

BITC — 2.5 5 10 — — — 5 10 5 10
SOR — — — — 2.5 5 10 5 5 10 10
KDa
Cyclin B1
β-Actin
60
43

MDA-MB-231

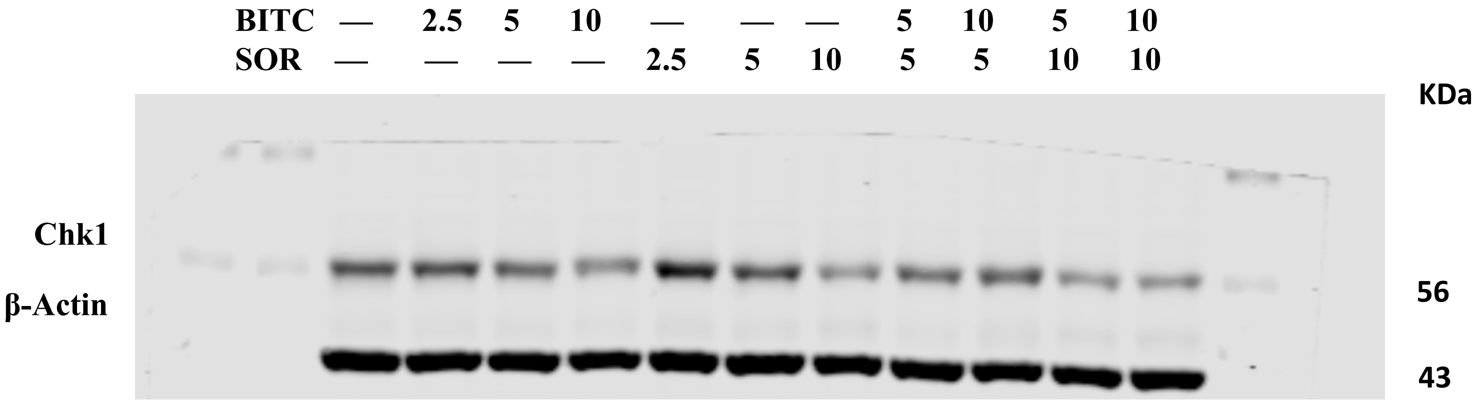

MDA-MB-231

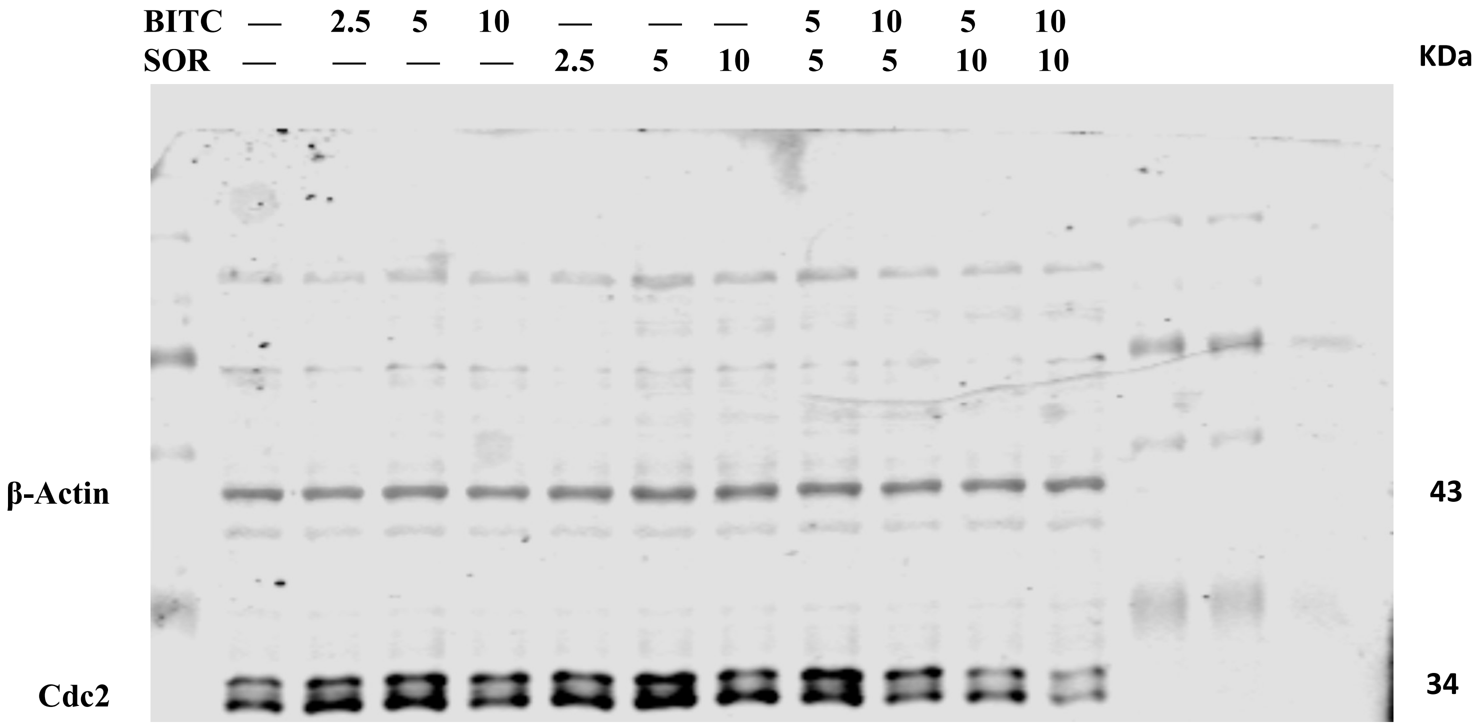

MDA-MB-231

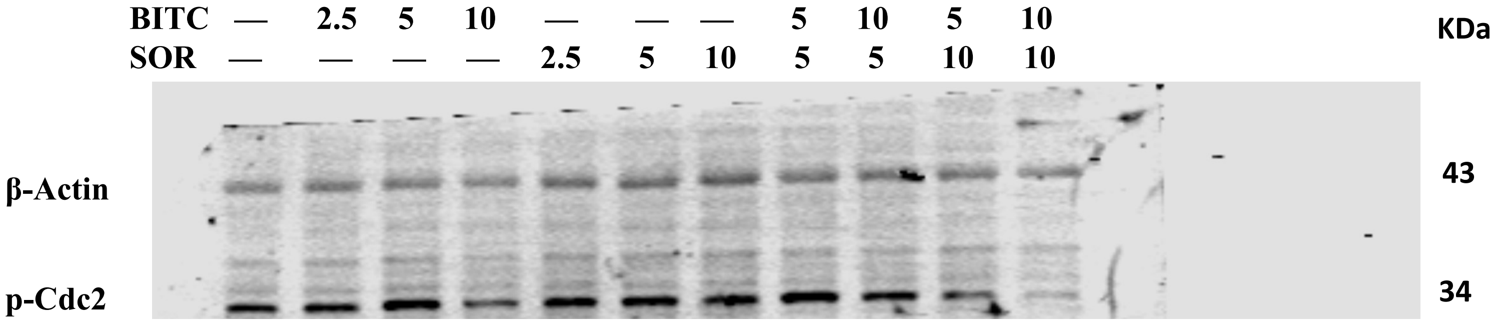

MCF 7

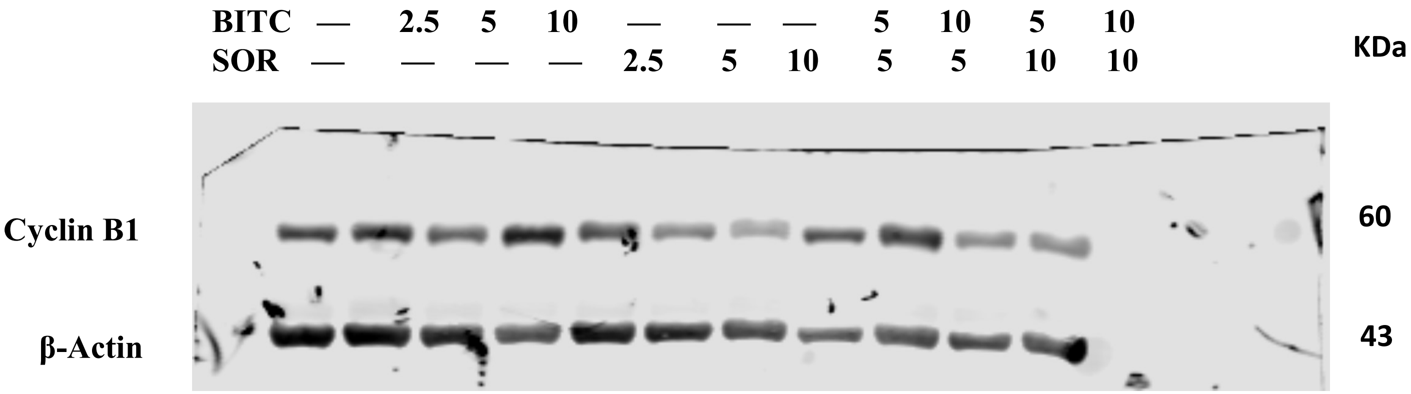
BITC — 2.5 5 10 — — — 5 10 5 10
SOR — — — — 2.5 5 10 5 5 10 10
KDa
Cyclin B1
60
β-Actin
43

MCF 7

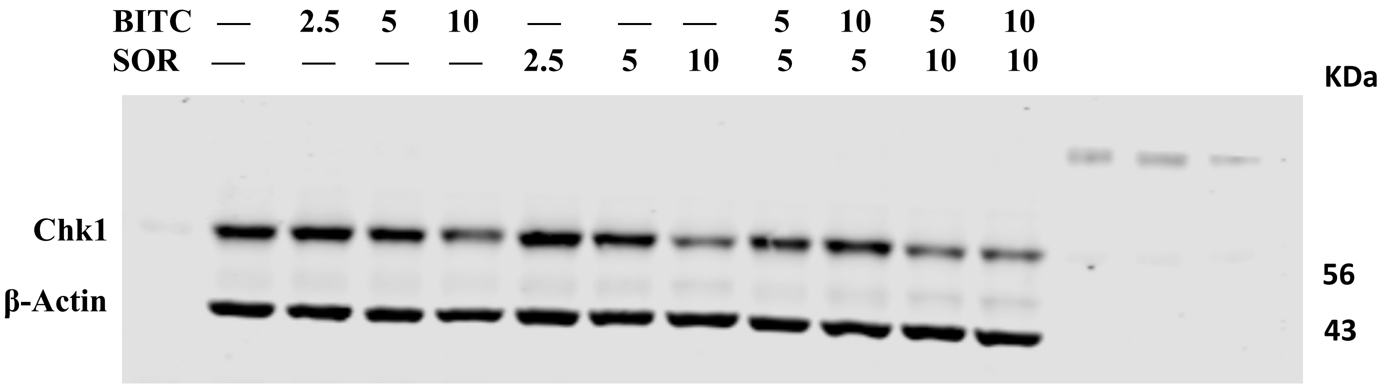

MCF 7

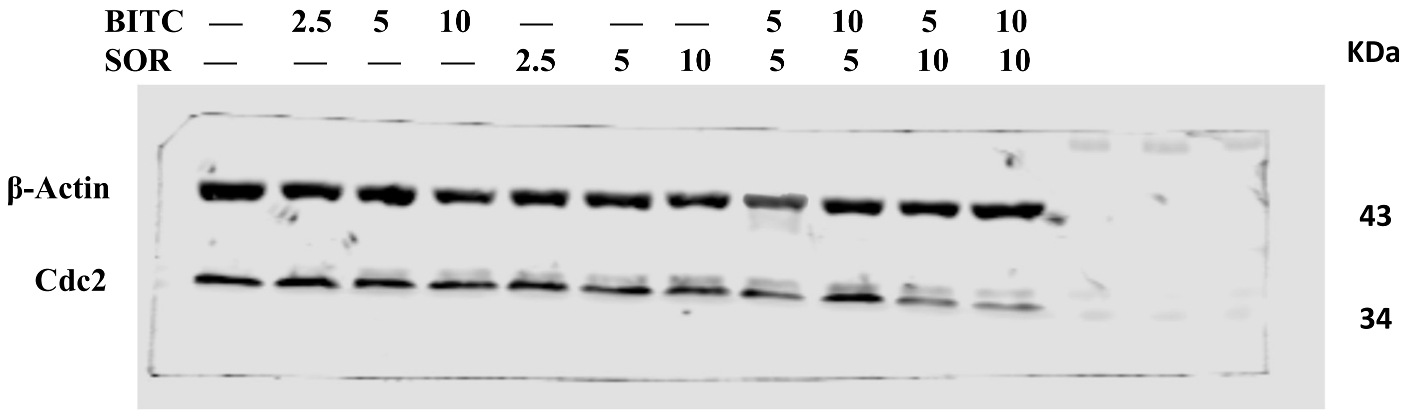

MCF 7

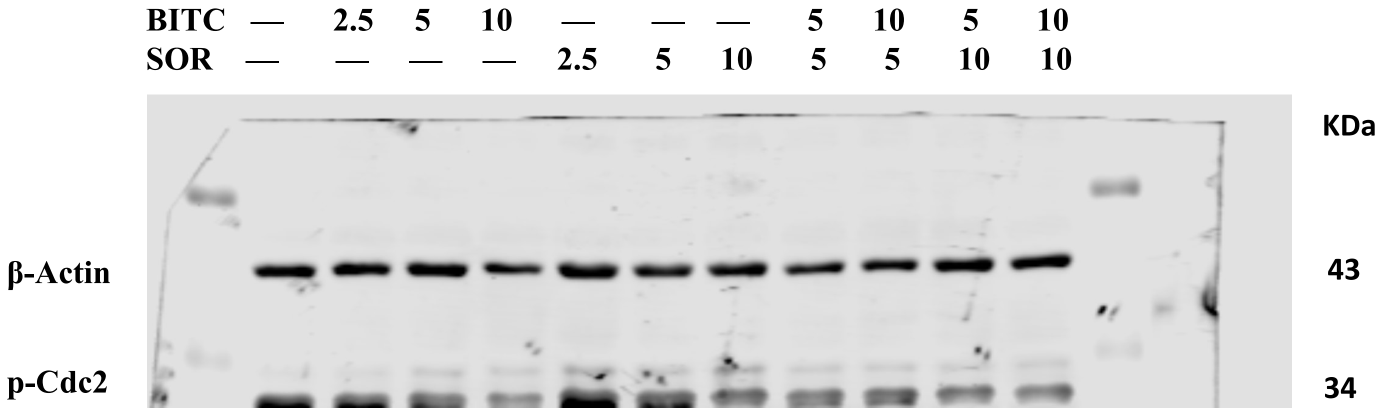

Supplement: Supplementary file 1 [file cancers-16-01695-s001.zip › cancers-2962962-File S1.pdf]
