# Supplementary material for: Synergistic Action of Benzyl Isothiocyanate and Sorafenib in a Nanoparticle Delivery System for Enhanced Triple-Negative Breast Cancer Treatment
Source: Cancers (Basel). 2024 Apr 26;16(9):1695. doi: 10.3390/cancers16091695 (PMC11083210; doi:10.3390/cancers16091695)
Supplement: Supplementary file 1 [file cancers-16-01695-s001.zip › cancers-2962962-supplementary.pdf]

Supplementary information:

Synergistic Action of Benzyl Isothiocyanate and Sorafenib in a Nanoparticle Delivery System for Enhanced Triple-Negative Breast Cancer Treatment

Qi Wang1*†, Nan Cheng1†, Wei Wang1, Yongping Bao1*

1 Norwich Medical school, University of East Anglia, NR4 7UQ, United Kingdom

* Correspondence: QW: q.wang1@uea.ac.uk, YPB: y.bao@uea.ac.uk

† These two authors contributed equally to this work

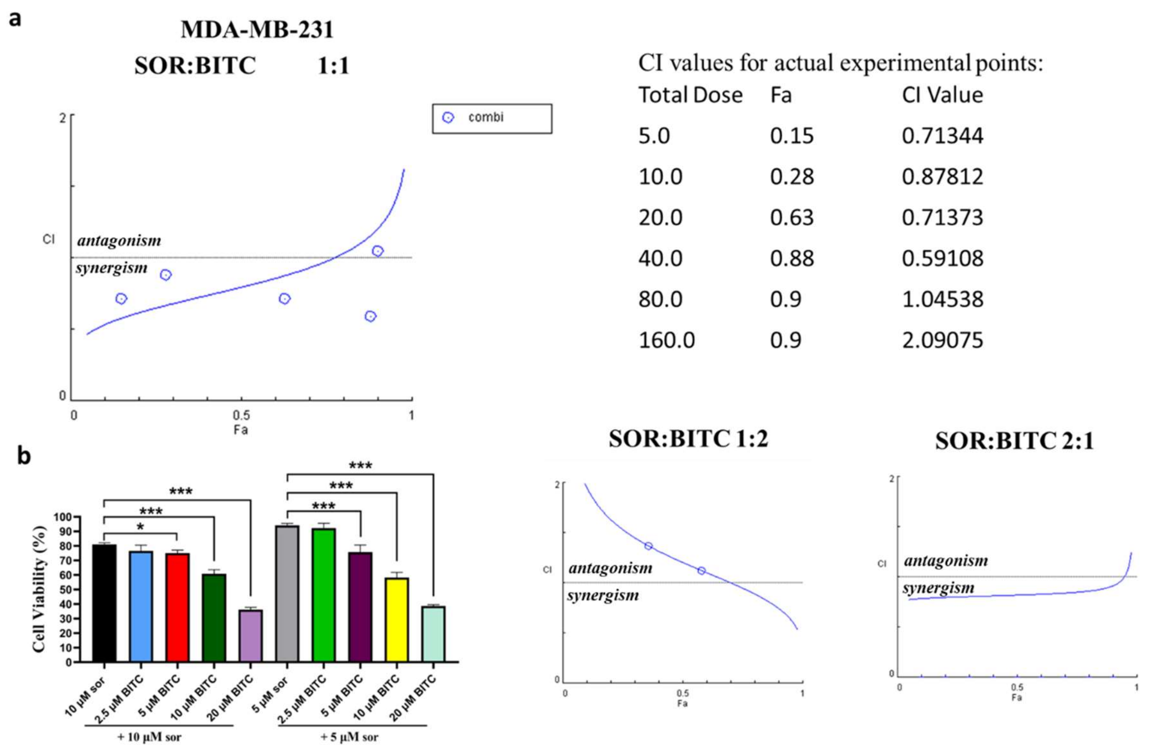

Figure S1. Chou-Talalay's Combinational Index (CI) was calculated using cell viability assay value in MDA-MB-231 cells treated with BITC and SOR combinations. (a) Combination Index Plot of BITC and SOR combination treatment. (Total Dose: dose of BITC plus dose of SOR; Fa: average effect values; CI value: combinational index value. CI values for synergism is 0-1, and for antagonism is 1-∞) (b) Cell viability of MDA-MB-231 cells was measured using MTT assays 24 hours after BITC and SOR treatment.

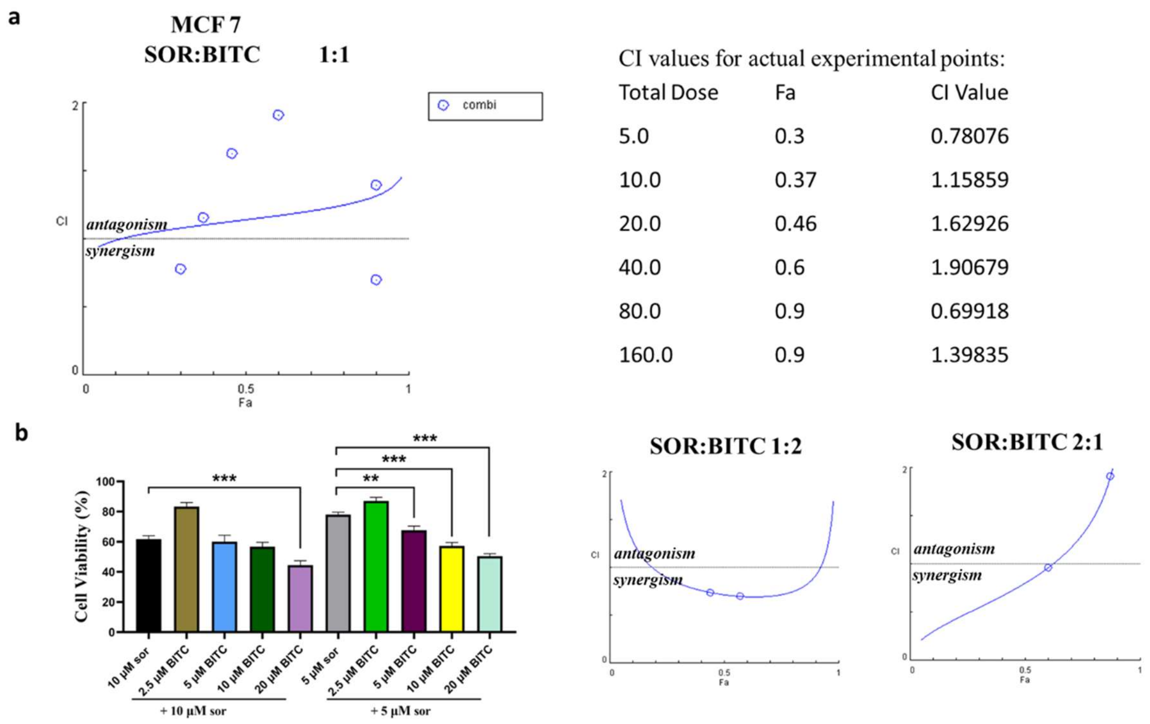

Total Dose	Fa	CI Value
5.0	0.3	0.78076
10.0	0.37	1.15859
20.0	0.46	1.62926
40.0	0.6	1.90679
80.0	0.9	0.69918
160.0	0.9	1.39835

Figure S2. Chou-Talalay's Combinational Index (CI) was calculated using cell viability assay value in MCF-7 cells treated with BITC and SOR combinations. (a) Combination Index Plot of BITC and SOR combination treatment. (Total Dose: dose of BITC plus dose of SOR; Fa: average effect values; CI value: combinational index value. CI values for synergism is 0-1, and for antagonism is 1-∞) (b) Cell viability of MCF-7 cells was measured using MTT assays 24 hours after BITC and SOR treatment.

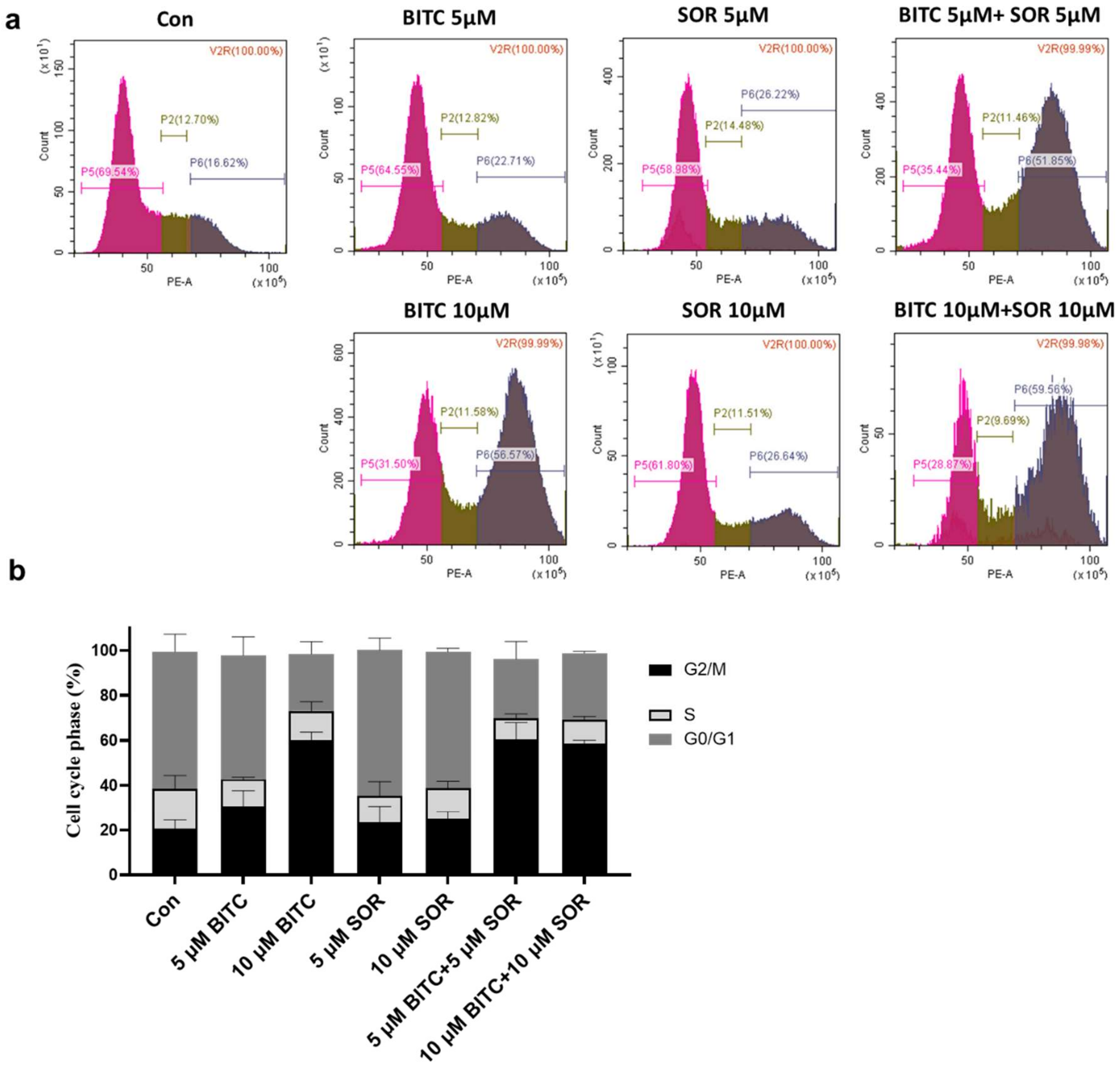

Figure S3. Effects of BITC and SOR combined treatment on MCF-7 cell cycle. (a) Flow cytometry analysis of the cell cycle distribution after 24 hours of treatment with BITC and/or SOR. (b) Quantitative evaluation of cells arrested at G2. Data are presented as means ± SD (n = 3). *P < 0.05, **P < 0.01, ***P < 0.001.

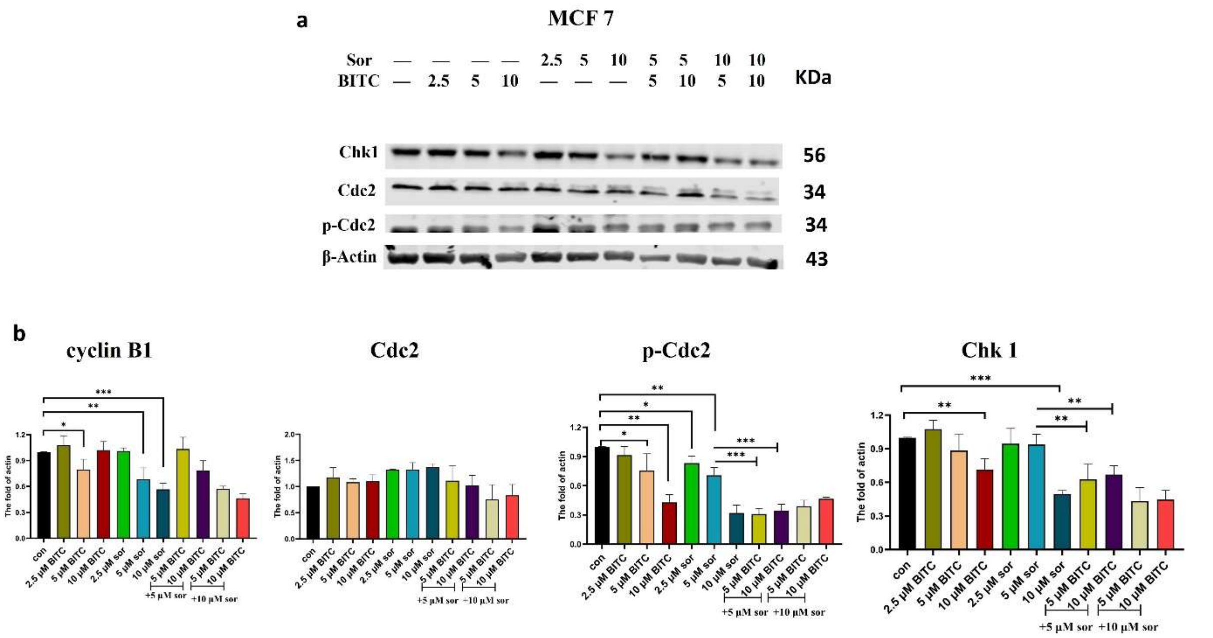

Figure S4. Effects of BITC and SOR treatment on G2 phase protein expression in MCF-7 cells. 24 hours after treatment, the expression of cyclin B1, Chk1, Cdc2, and p-Cdc2 proteins was analyzed by Western blot. Expression of β-Actin served as a loading control. Band densities were normalized against β-actin, and results were expressed as fold changes relative to controls. Data are presented as means ± SD (n = 3). *P < 0.05, **P < 0.01.

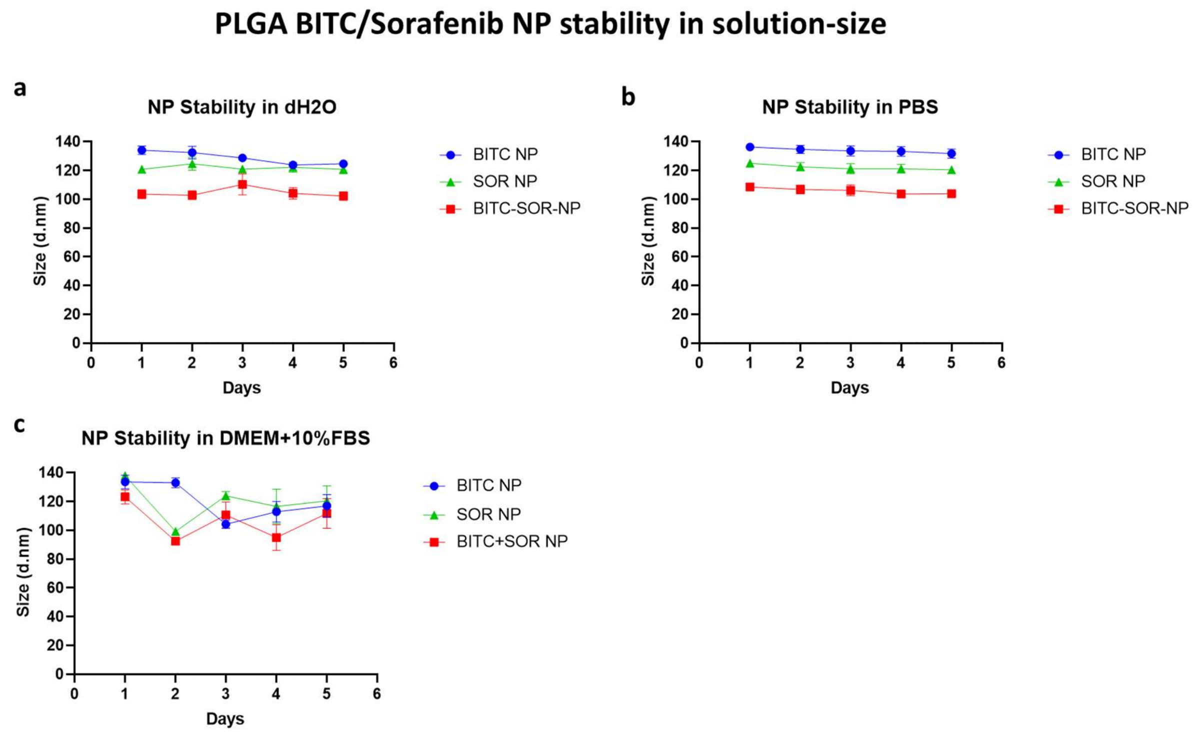

Figure S5. BITC and SOR encapsulated nanoparticle size stability in various solutions. (a) dH_2O, (b) PBS, (c) DMEM+10%FBS. The size of nanoparticles was measured by dynamic light scattering.

PLGA BITC/Sorafenib NP stability in solution-polydispersity index

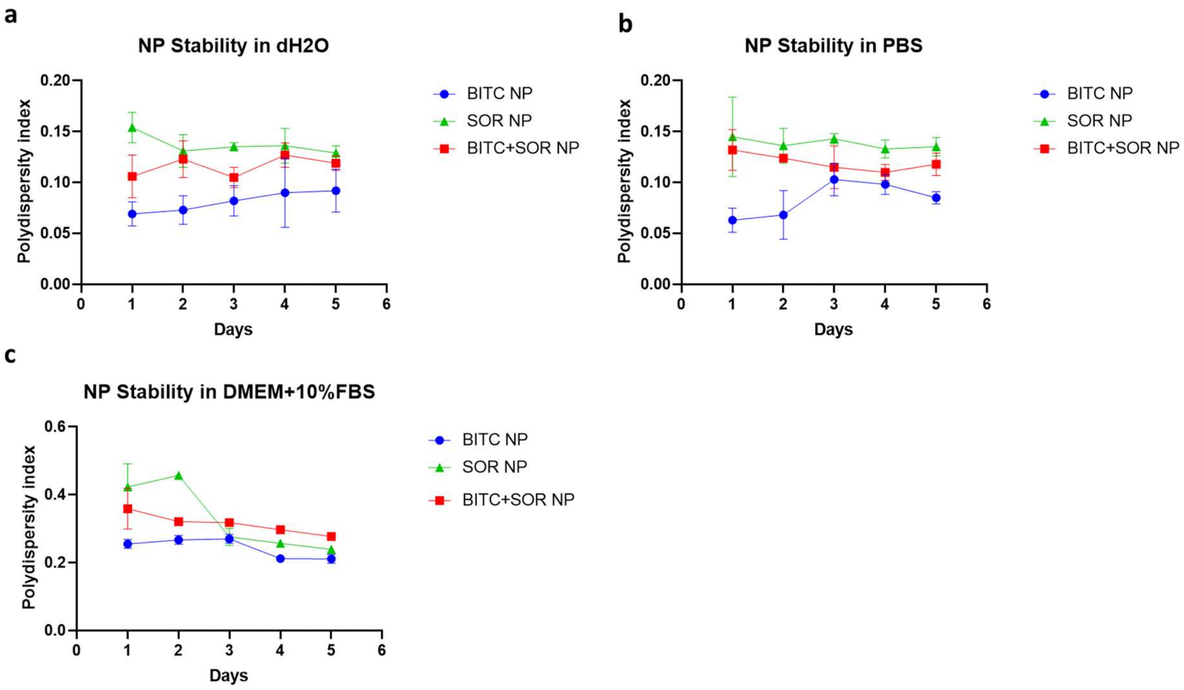

Figure S6. BITC and SOR encapsulated nanoparticle Polydispersity index (PDI) stability in various solutions. (a) dH_2O, (b) PBS, (c) DMEM+10%FBS. The PDI of nanoparticles was measured by dynamic light scattering.

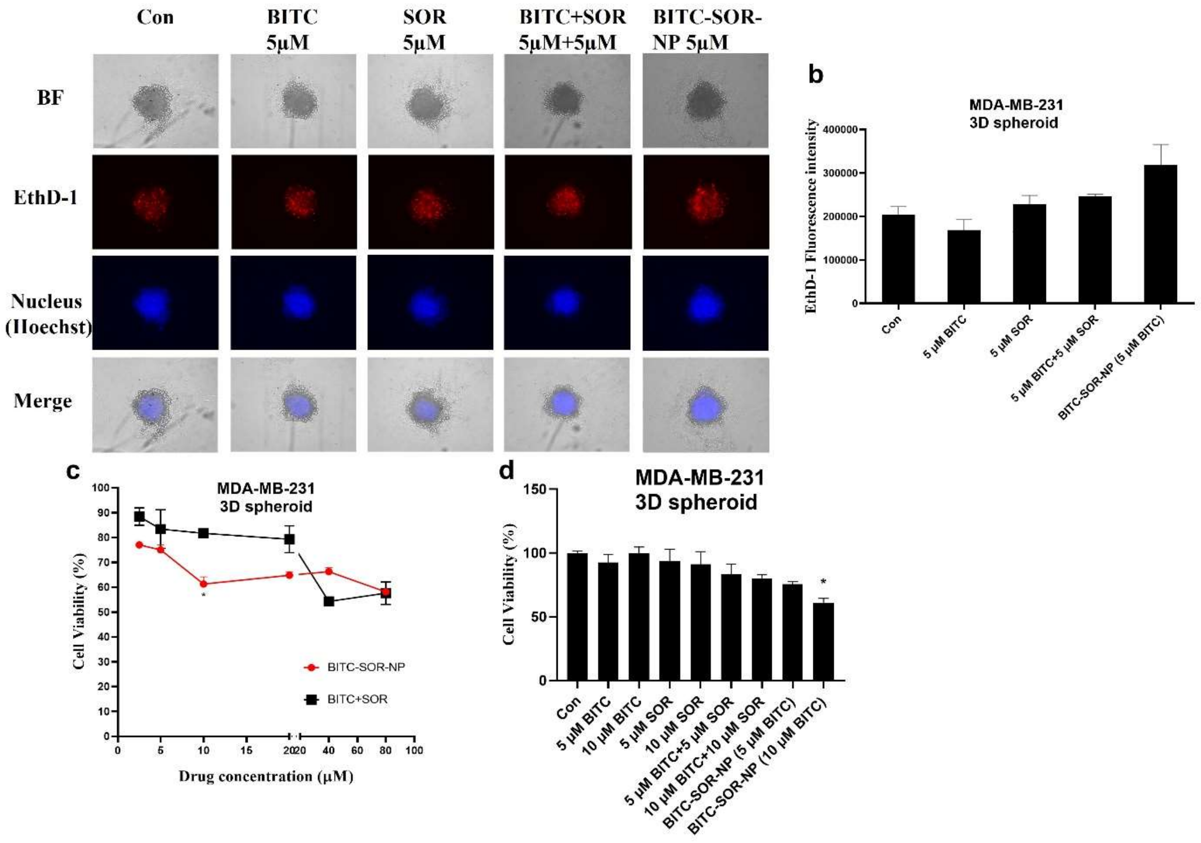

Figure S7. Effects of BITC-SOR-NPs treatment on 3D spheroid of MDA-MB-231 spheroids. (a) Cytotoxicity of MDA-MB-231 spheroids after BITC and/or SOR free drugs or encapsulated NP treatments for 4 days. Representative images of MDA-MB-231 spheroids were captured using EVOS M5000 microscope under 10x magnification brightfield image, nuclear (blue Hoechst dye staining) and dead (red EthD-1 dye staining) cell population. (b) Fluorescence intensity of EthD-1 dye staining on MDA-MB-231 spheroids after BITC and/or SOR treatments. (c) and (d) Cell viability study of MDA-MB-231 spheroids after 48h treatments of BITC-SOR-NPs and free drug combination. Data are presented as means ± SD (n = 3). *P < 0.05.

Table S1: Table of PLGA BITC/Sorafenib NP characteristics

	**Size (d.nm)**	**Polydispersity Index (PdI)**	**BITC Encapsulation efficiency (BITC-EE%)**	**Sorafenib Encapsulation efficiency (Sor-EE%)**	**Zeta potential (mV)**
**BITC NP**	136.3±1.4	0.063±0.012	66.2±6.0	NA	-12.0±0.4
**SOR NP**	125.0±0.8	0.145±0.039	NA	42.6±2.1	-12.1±0.1
**BITC-SOR-NP**	108.6±1.9	0.132±0.020	24.0±1.5	38.4±6.3	-7.6±0.3
**ENP**+	93.8±0.7	0.137±0.036	NA	NA	-5.0±0.7

+ ENP: empty NP without drug encapsulation
